# Unraveling the Dysbiosis of Vaginal Microbiome to Understand Cervical Cancer Disease Etiology—An Explainable AI Approach

**DOI:** 10.3390/genes14040936

**Published:** 2023-04-18

**Authors:** Karthik Sekaran, Rinku Polachirakkal Varghese, Mohanraj Gopikrishnan, Alsamman M. Alsamman, Achraf El Allali, Hatem Zayed, George Priya Doss C

**Affiliations:** 1 School of Biosciences and Technology, Vellore Institute of Technology, Vellore 632014, India; 2 Molecular Genetics and Genome Mapping Laboratory, Genome Mapping Department, Agricultural Genetic Engineering Research Institute, Cairo 12619, Egypt; 3 African Genome Center, Mohammed VI Polytechnic University, Ben Guerir 43150, Morocco; 4 Department of Biomedical Sciences, College of Health Sciences, QU Health, Qatar University, Doha 2713, Qatar

**Keywords:** cervical cancer, eXplainable AI, vaginal microbiome, SHapley Additive exPlanations

## Abstract

Microbial Dysbiosis is associated with the etiology and pathogenesis of diseases. The studies on the vaginal microbiome in cervical cancer are essential to discern the cause and effect of the condition. The present study characterizes the microbial pathogenesis involved in developing cervical cancer. Relative species abundance assessment identified *Firmicutes*, *Actinobacteria*, and *Proteobacteria* dominating the phylum level. A significant increase in *Lactobacillus iners* and *Prevotella timonensis* at the species level revealed its pathogenic influence on cervical cancer progression. The diversity, richness, and dominance analysis divulges a substantial decline in cervical cancer compared to control samples. The β diversity index proves the homogeneity in the subgroups’ microbial composition. The association between enriched *Lactobacillus iners* at the species level, *Lactobacillus*, *Pseudomonas*, and *Enterococcus* genera with cervical cancer is identified by Linear discriminant analysis Effect Size (LEfSe) prediction. The functional enrichment corroborates the microbial disease association with pathogenic infections such as aerobic vaginitis, bacterial vaginosis, and chlamydia. The dataset is trained and validated with repeated k-fold cross-validation technique using a random forest algorithm to determine the discriminative pattern from the samples. SHapley Additive exPlanations (SHAP), a game theoretic approach, is employed to analyze the results predicted by the model. Interestingly, SHAP identified that the increase in Ralstonia has a higher probability of predicting the sample as cervical cancer. New evidential microbiomes identified in the experiment confirm the presence of pathogenic microbiomes in cervical cancer vaginal samples and their mutuality with microbial imbalance.

## 1. Introduction

Cancer is a major contributor to mortality and a significant impediment to extending life expectancy. Global predictions indicate that the burden of cancer will increase for at least the next two decades, contributing significantly to the burden of illness [1,2]. Reproductive malignancies constitute a significant cause of female mortality and morbidity worldwide. Cervical cancer is more prevalent in the female reproductive system malignancies, with 569,847 cases per year, ranking it fourth among the malignancies that strike women globally [2,3]. Cervical cancer initially develops in the cervix uteri, and the malignancy transpires slowly overtime.

The key detrimental factor for the preponderance of cervical cancer is exposure to sexually transmitted human papillomavirus (HPV) [4]. If identified at its initial stages, cervical cancer may be one of the most treatable forms of cancer [5]. The problem is that most patients only seek therapy once the disease has progressed to a late stage. Many potential reasons exist for patients with cervical cancer to seek treatment at a later stage and have a poor prognosis. The paucity of knowledge, cultural issues, the absence of coordinated cancer prevention, as well as inadequate HPV vaccination strategies are a few reasonable factors [6].

HPV infection is a predominant cause of cervical cancer; environmental factors might also significantly impact cancer progression. Epidemiological studies have repeatedly identified smoking as contributing to cervical cancer [7,8]. The microbial communities are one of the elements yet to be substantially researched. The etiology of cervical cancer is multifaceted, and there is less scientific evidence to support the involvement of bacterial groups in cervical carcinogenesis [9,10]. Although microbial diversity is perceived as a sign of health across different body sites, highly diversified vaginal microbiomes are prominently viewed as aberrant or dysbiotic and usually linked to a diseased condition [11,12]. The metagenomic concepts and the transition of high-throughput sequencing analysis have sparked interest in the connection between microbes and various diseases. According to a study by Huang et al., 2014, vaginal microbiome plays a significant role in preserving vaginal homeostasis and limiting the growth of dangerous bacteria [13].

Recent research has evaluated the potential link between cervical cancer and vaginal microbiome [14,15,16,17,18,19]. Cervical microbiome varies from person to person [20]. It is being investigated as a target for developing novel treatment methods due to mounting evidence that it plays a significant role in the uterine cervix’s carcinogenesis process [21,22]. The cervical microbiome is crucial as it possesses the metabolic and enzymatic machinery needed to digest vital vitamins, eliminate harmful substances, fight off infections, support the female genital tract epithelium, and activate and control the immune system [23]. According to earlier research, changes occurred in the cervical microbiota, enhancing the likelihood of carcinogenic development in the cervix. Similar studies demonstrated that altering the cervical microbiome increases the risk of carcinogenic progression [24,25,26]. Despite the intriguing antecedent results published up to this point, little is still understood about the intricate relationship between cervical dysbiosis and cancer pathogenesis. There is a critical need to compare differences in women with different grades of cervical cancer and their microbial composition to fully understand the microbiome actively involved during cervical cancer pathogenesis. The present study analyzes the vaginal microbial samples of cervical cancer and control groups. Abundance assessment at different taxonomic levels is performed. The α and β diversity are calculated with richness, dominance, and similarity indices of microbial communities between groups. LEfSe analysis detected enriched microbiomes at an LDA score threshold of 3.0. Further, the functional enrichment predicted highly correlated disease association based on the differential microbiomes. SHAP algorithm interpreted the random forest predictions to understand specific microbiomes influencing the results.

## 2. Materials and Methods

### 2.1. Data Acquisition

This study intends to compare and analyze the dysbiosis in the vaginal microbiome of cervical cancer patients and healthy individuals. “Cervical cancer” and “Vaginal microbiome” keywords were used to search the NCBI BioProject by applying the filters “Human” as the organism type and “metagenome” as the study type. The vaginal swab samples collected from cervical cancer patients and healthy individuals were sequenced using the 16S rRNA technology to create the final dataset (BioProject ID: PRJNA725946). The vaginal samples were extracted from the genomic DNA using QIAamp DNA Mini Kit and processed with Illumina HiSeq platform at Dalian Medical University, Dalian, China. The samples were labeled according to the patients and the controls. The dataset comprises 65 cervical cancer samples and 54 healthy samples collected using a vaginal swab.

### 2.2. Bioinformatic Processing and Statistical Analysis

The raw FASTQ files for the vaginal samples (BioProject ID: PRJNA725946) were retrieved from the European Nucleotide Archive (ENA). The single-end reads fetched from the 16S rRNA sequencing method were perused using Quantitative Insights into Microbial Ecology version 2 (QIIME2 v. 2022.8) (https://qiime2.org/ (accessed on 4 December 2022)) [27]. The single-end reads were imported into the QIIME2 and demultiplexed to check the quality of reads. The low-quality reads (Q < 30) were eliminated from the pipeline using trimming and truncation methods. For the single-end reads, the trimming was performed at a beginning position of 0 and abridged at a base length of 240 bp. The DADA2 algorithm was further used to locate and eliminate the chimeric sequences. Following the conventional DADA2 workflow with modifications to accommodate our single-end read data, the 16S sequences were denoised [28].

The sequence’s lowest bound of the sampling depth (24,217) was identified to keep all the samples. The sequences with more than 99% similarities were considered Amplicon Sequencing Variants (ASVs). The ASVs considered less than 0.001% of the overall abundance were eliminated to ensure the correctness of the subsequent analysis [29]. The species-level designations were based on precise matching between ASVs and the sequenced reference strain; the taxonomy was determined using the Naïve Bayesian classifier approach using the 16S Silva database (silva-138-99-nb-classifier v. 13_8) [30]. After the aforementioned preprocessing steps, sequences from the phyla of mitochondria and chloroplast were disregarded, as well as those from the kingdoms of Archaea and Eukaryota [31]. The resultant QIIME data, such as the feature and taxonomy tables, were subjected to statistical analysis.

The heterogeneity and uniformity of the microbiota among cervical cancer-affected cases and healthy women were evaluated using α and β diversity analysis [32]. Sequences from each sample were rarefied to a depth of 24,217 to perform the diversity analysis [33]. The samples’ α diversity analysis was evaluated using Chao1, Shannon, and Simpson measures based on Wilcoxon rank-sum test [34]. The species differences between the samples were computed using β diversity analysis (PCoA) with Bray Curtis distance metric [35]. The visualization plots for the abovementioned analysis were generated using the micro eco R package [36]. The coalition network was constructed with the igraph R package [37]. Using methods from the igraph package, topographical network characteristics such as centrality and edge weights were also examined.

The differentially represented microbial species between groups at different levels in the taxonomic scale were determined using the LEfSe (http://huttenhower.sph.harvard.edu/galaxy/ (accessed on 5 December 2022)). LDA employs the Kruskal–Wallis approach to determine the traits that show differential abundance among various classes. Using the LEfSe method, variations in microbial abundance between diseased and healthy control groups were determined with a logarithmic LDA score of 4.0. A cladogram and bar graph drawn to show the taxonomic traits are the outputs of the LEfSe model [38]. The functional disease enrichment was performed using the R package MicrobiomeProfiler to study the association between vaginal microbiome and cervical cancer. The microbe–disease enrichment analysis module from the package was utilized to perform the enrichment analysis.

### 2.3. SHAP Interpretation of Vaginal Microbiome Associated with Cervical Cancer

The collapsed taxonomic table at the species level containing ASVs and taxa information of all the samples was processed with the “DALEX” library in Python [39]. This analysis was intended to show the species identified to have a strong association with cervical cancer alongside complete taxonomic information. SHAP (Shapley Additive Explanations) and DALEX (Descriptive Machine Learning Explanations) are two popular Python libraries used for explainable artificial intelligence (XAI) [40,41]. These libraries provide tools for understanding the behavior of complex machine learning models, such as deep neural networks, decision trees, random forests, and gradient-boosting machines. In this experimental work, the interpretability of the random forest algorithm was evaluated on the vaginal microbial data.

SHAP is a game theoretic approach to explain the output of any machine learning model. It aims to explain the contribution of each input feature to the final model prediction. SHAP computes the Shapley values, which is a measure of the marginal contribution of a feature towards the prediction. Shapley values provide a unified framework for explaining any machine learning model, regardless of its complexity. SHAP also provides visualizations that help understand each feature’s importance in the model output. DALEX explains the behavior of machine learning models with the help of visualizations. It provides tools for model-agnostic explanations, feature importance, and model diagnostics.

## 3. Results

To compare the vaginal microbiome differences between the cervical cancer patients and healthy controls using the ASVs, 119 metagenome sequenced samples were retrieved from the cervical cancer study, including 65 cervical cancer patients (54.6%) and 54 healthy controls (45.3%).

### 3.1. Characterization of Vaginal Microbiome

**Figure 1:**
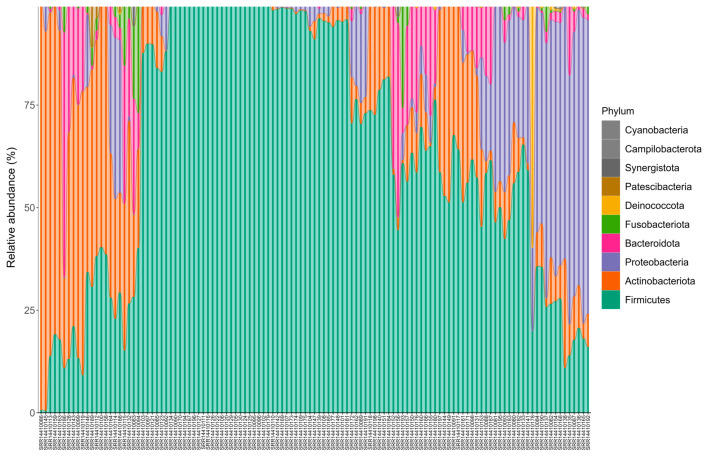
Alluvial plot depicting the taxonomic abundance of ASVs associated with the samples at a phylum level.

**Figure 2:**
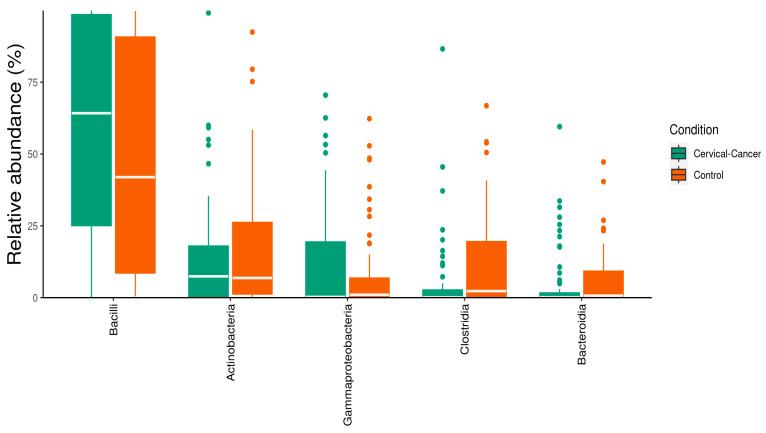
Boxplot illustrating the relatively abundant microbiome of diseased and control groups at the class level. The cervical cancer group is shown in green color, and the healthy control group is shown in orange color.

**Figure 3:**
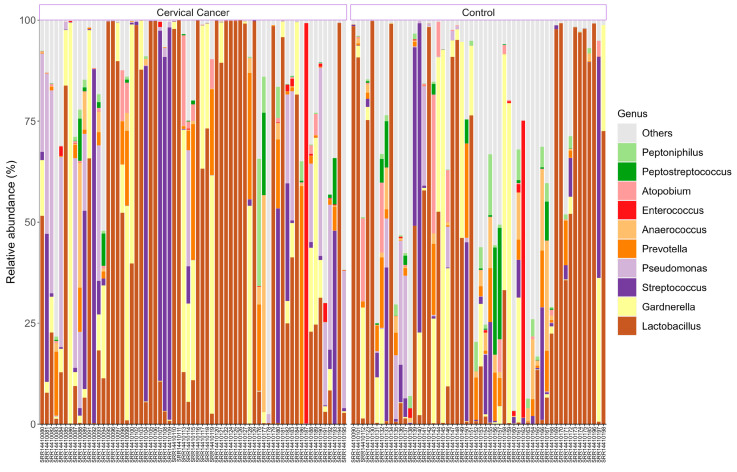
An illustration of a bar plot showing the relative abundance of diseased and control groups at the genus level (cervical cancer—**left**; healthy control—**right**).

**Figure 4:**
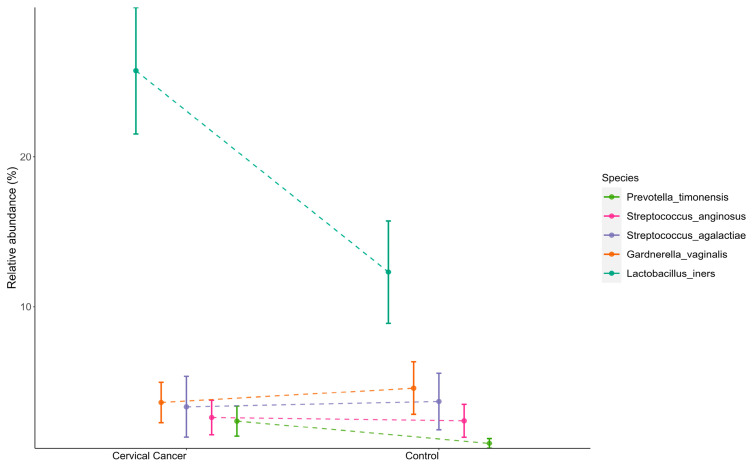
Line plot representing the taxonomic abundance at species level across the different groups (cervical cancer—**left**; healthy control—**right**).

After the quality filtering process, there were 5,253,668 reads with a mean value of 44,148 reads per sample. In total, 1973 ASVs were detected after clustering for the sequences at a 99% similarity with the SILVA database. The mean taxon abundance was assessed at different taxonomic levels, such as species, genus, family, class, and phylum, for both cervical cancer and control groups. The top five bacteria belonged to *Firmicutes*, *Actinobacteriota*, *Proteobacteria*, *Bacteroidota*, and *Fusobacteria*, with *Firmicutes* being the most predominant phyla in both groups (Figure 1). The higher taxonomic abundancies at the class level were observed in *Bacilli*, *Actinobacteria*, *Gammaproteobacteria*, *Clostridia*, and *Bacteroidia,* of which *Bacilli* showed greater prevalence (Figure 2). In terms of abundance, Lactobacillus was shown to be the most prevalent, followed by *Gardnerella*, *Streptococcus*, and *Pseudomonas* at the genus level (Figure 3). No cardinal variations were observed in abundance between cervical cancer and healthy control groups at the genus level. *Lactobacillus iners*, *Gardnerella vaginalis*, *Streptococcus agalactiae*, *Streptococcus anginosus*, and *Prevotella timonensis*, among which *Lactobacillus iners* showed higher preponderance in the cervical cancer group at the species level (Figure 4).

### 3.2. Dysbiosis of Vaginal Microbiome Associated with Cervical Cancer

**Figure 5:**
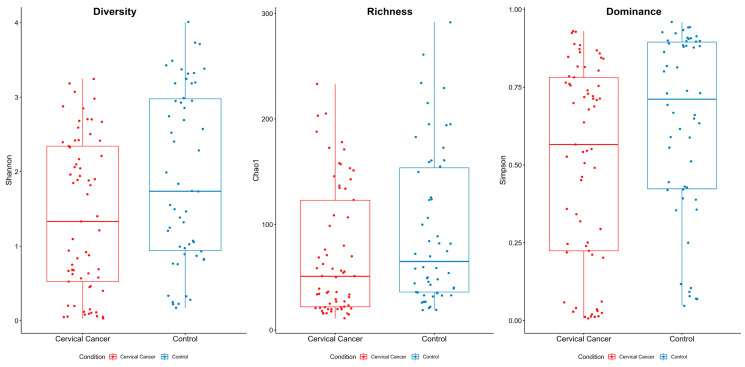
α diversity indexes are plotted as boxplots. α diversity indices are composite indices that capture consistency and abundance. The Shannon and Simpson indices reflect ASV diversity in samples, and the Chao1 measure reflects the ASV abundance in samples.

Simpson, Shannon, and Chao1 indices were used to understand the complexity of species heterogeneity between the two groups. The species richness within the samples can be reflected using Chao1, whereas Shannon and Simpson indices depict the species diversity within a community (species richness and diversity). The Chao1 measure is considerably higher for healthy control than for the cervical cancer group. As per the findings, species richness is substantially higher in healthy controls. The Shannon and Simpson measures show higher indices for the healthy control group than the cervical cancer group (Figure 5).

**Figure 6:**
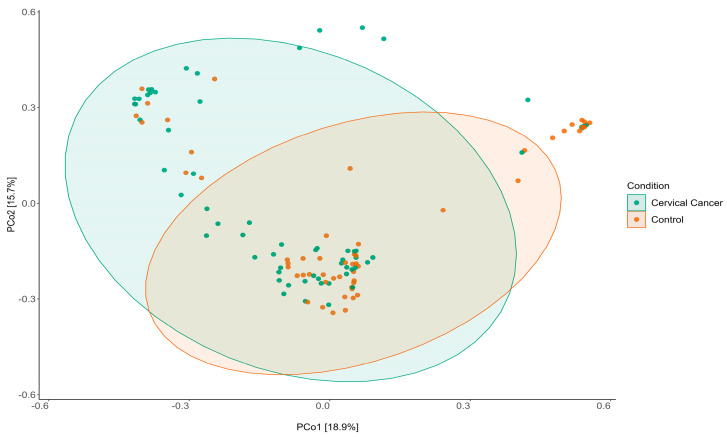
PCOA plots of β diversity of vaginal microbiota based on Bray−Curtis distance measure. The ellipses represent the two groups. The cervical cancer is shown in green color, whereas the healthy control group is shown in orange color.

The vaginal microbiota diversity among the two groups was compared using the Bray–Curtis distance measure. The microbial makeup of each group can be represented using a Principal coordinate analysis (PCoA) plot (Figure 6). In PCOA plots, the samples closer to each other resemble similar microbial communities. In the PCoA plot, the two coordinates (PCo1 and PCo2) account for 34.7% of the variation.

**Figure 7:**
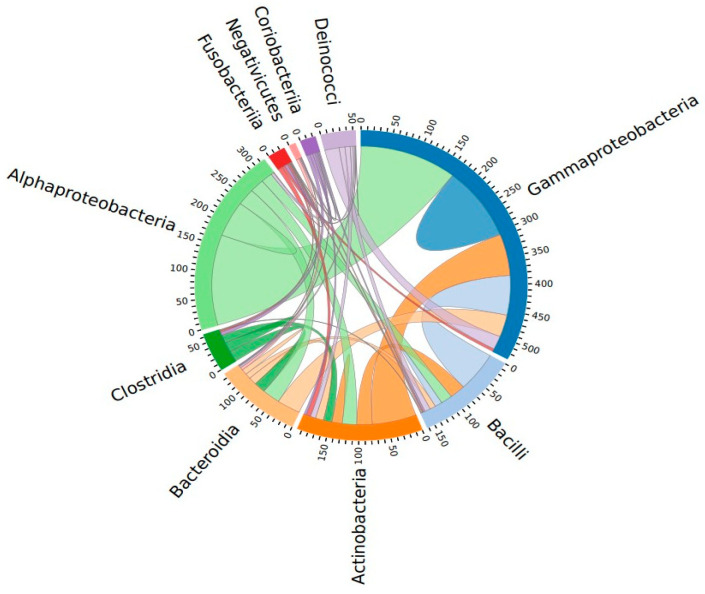
The chord diagram displays the network of 10 candidates that co-occur in a pairwise sequence. Each sector of the circle represents a node (i.e., taxon) in the network, and its width reflects the sum of the co-occurrences between each taxon.

The coalition network can be used to depict the associativity between microorganisms present within a group or a community. The PCoA plot indicates a significant distinction among the vaginal microbial communities of cervical cancer and healthy control groups (*p*-value: 0.001, R2: 0.027, F-value: 3.269). The igraph bipartite approach was used to identify the connections among different microbes at the class level. Alphaproteobacteria were identified as the key taxon within the network that formed pairwise co-occurrence networks with the other microbes, particularly with *Gammaproteobacteria*, *Bacteroidia*, *Actinobacteria*, and *Bacilli* (Figure 7).

**Figure 8:**
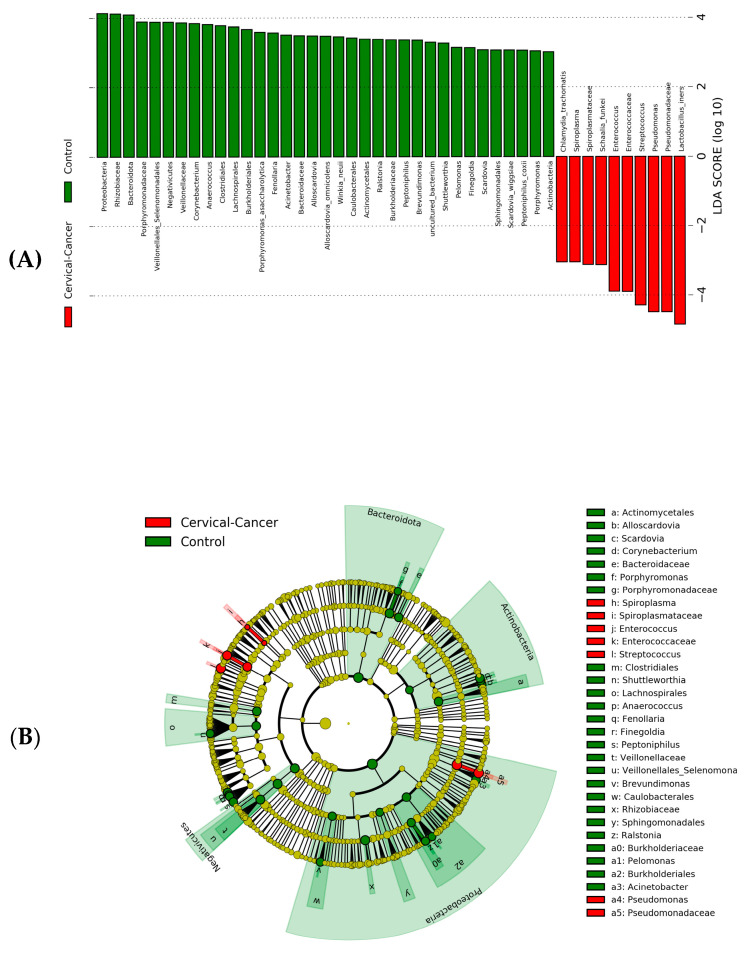
Linear discriminant analysis (LDA) effect size (LEfSe) analysis for vaginal microbiota abundance in cervical cancer and healthy control groups. (**A**) The Bar plot from LEfSe analysis indicates the enriched bacteria that are associated with cervical cancer (red) and healthy control (green) groups. (**B**) A phylogenetic cladogram plot from LEfSE analysis representing the differentially abundant taxa at various taxonomic levels (LDA score > 3.0).

LEfSe assessment identifies the microbial abundance of cervical cancer patients and healthy control group from the vaginal microbiome. The LEfSe profiling shows variations between cervical cancer and healthy control groups at various taxon levels with a threshold LDA core of 4.0 (Figure 8). In cervical cancer patients, the cladogram shows a significant abundance of *Lactobacillus iners*, *Pseudomonadaceae*, *Enterococacceae*, and *Entomoplasmatales*, whereas *Proteobacteria*, *Actinobacteria*, and *Bacteroidota* are displayed in the healthy control (Figure 8).

**Figure 9:**
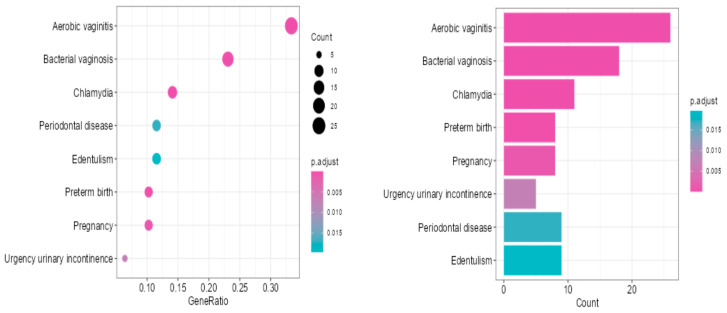
Comparative disease–microbiome enrichment analysis of vaginal microbiota depicted as line plot and bar plot. A total of 77 significantly different bacterial taxa were reported in the enrichment analysis.

The differential expressed taxa were detected using MicrobiomeProfiler to identify the bacterial strains enriched in the vaginal microbiota of cervical cancer patients. The disbiome database was selected for microbiome disease enrichment analysis, for which the taxon IDs of identified bacterial strains (135) were provided as input (Table S1). The microbial strains were determined to be associated with eight diseases, of which the microbial enrichment were highly associated with Aerobic vaginitis, Bacterial vaginosis, and Chlamydia, respectively. The functional enrichment outcome between cervical cancer and healthy vaginal microbiome samples is represented in Figure 9.

### 3.3. Explaining the Model Predictions through SHAP

Interpreting “black-box” mathematical models is pivotal to understanding complex biological outcomes. Traditional machine learning algorithms generate results based on intuitive, logical assessments derived through mathematical models. However, the reason for every model prediction is unknown due to the higher level of abstraction and deeper computing process. It is also arduous to analyze each step of interminable calculation performed by the algorithms. Explainable Artificial Intelligence (XAI), a sophisticated algorithmic approach, was developed by the Defense of Advanced Projects Research Agency (DARPA). It is intended to develop self-explainable human understandable models while maintaining higher-level performance. Shapley Additive Explanations (SHAP), a game theoric approach-based framework, conduct interpretable predictions from the results of any trained machine learning model. This method assigns importance to a particular sample prediction variable based on the Shapley values. The average marginal contribution of every feature score over all other possible coalitions calculates it. DALEX provides tools for creating various model-agnostic explanations, such as feature importance plots, partial dependence plots, and accumulated local effects plots. The SHAP value plot, breakdown, and ROC curve results are visualized using DALEX.

**Figure 10:**
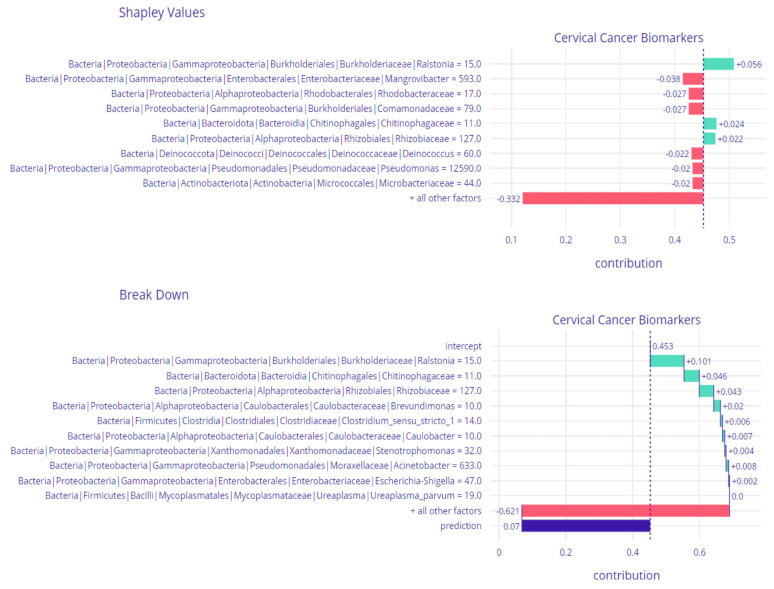
SHAP explanations for the cervical cancer group, the top bar plot depicts the importance of each feature in terms of SHAP values. The bottom plot represents the feature breakdown. The red bar signifies a declining pattern, whereas the green bar shows an increase in the average response for each feature.

**Figure 11:**
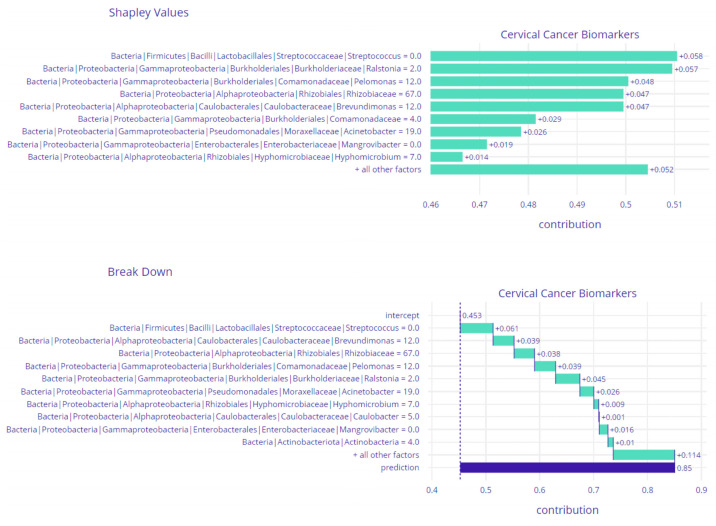
The SHAP explanations for the healthy control group, where the bar plots represent feature significance using SHAP values and feature breakdowns, respectively. The green patterns illustrate the substantial increase in average response for each feature.

The microbiome dataset contains a taxonomic hierarchy from Kingdom to Species-level of each column as a feature vector with 594 taxons in total, and 119 rows represent individual samples. Random forest, an ensemble-based bagging model, is trained with the data to numerically understand the discriminative pattern between microbiomes of cervical cancer and control samples. The model performance is evaluated through k-fold cross-validation (K = 10) and repeated k-fold cross-validation with five repeats. The k-fold and repeated k-fold CV scores are 0.926 and 0.971, respectively, and share no big difference between the results (Supplementary file). The resultant model of repeated k-fold CV is inputted into the SHAP model to understand the predictions. Two samples from the dataset of each study group are randomly drawn for interpretation. The SHAP results of cervical cancer and control samples are depicted in Figure 10 and Figure 11, respectively. The *X*-axis represents the taxonomic label, and the contribution of each feature is provided as a probability score in the *Y*-axis. The top bar plot of Figure 10 and Figure 11 visualizes the importance of each feature contributing to predicting a particular class in terms of SHAP values.

Similarly, the bottom bar plot provides each feature breakdown contributing to the correct prediction of the corresponding sample class. Each feature’s negative and positive impact on the predictions is represented in red and green. The green bar indicates the increase in the average response of each feature, whereas the red bar denotes the decreasing pattern. The intercept value is the average response score; in the current model, it is 0.453.

The increased *Ralstonia* at the genus level, *Chitinophagaceae*, and *Rhizobiaceae* Family level positively impacted the sample prediction as cervical cancer, provided at the top of Figure 10. The breakdown figure at the bottom provides the positive contribution of each microbiome in the prediction. This inference exhibits the importance of the microbiomes mentioned above in classifying cervical cancer individuals. The analysis of the control sample in Figure 11 determined that the decreased count of *Streptococcus*, *Ralstonia*, *Pseudomonas*, and *Brevundimonas* at the genus level positively correlated with the control sample.

**Figure 12:**
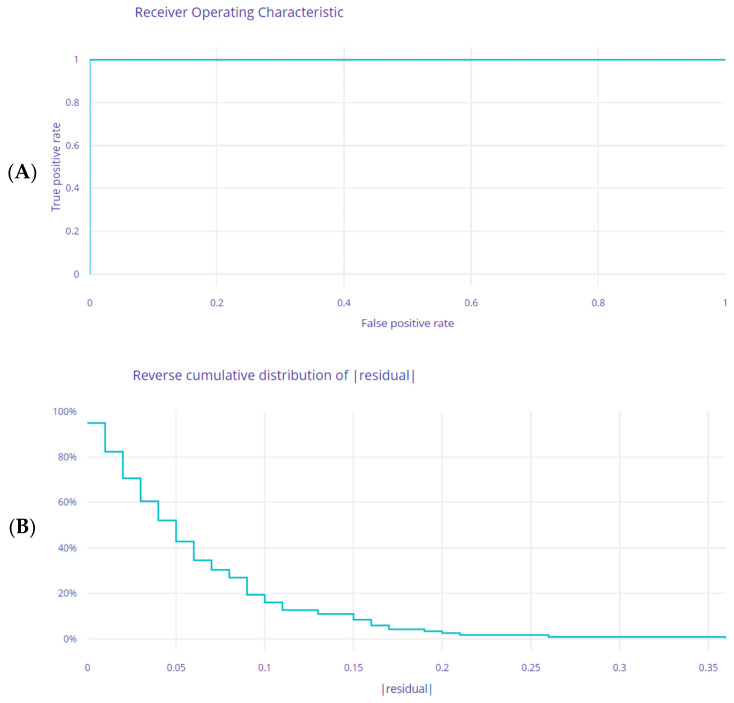
Model construction and feature screening using machine learning algorithms. (**A**) ROC curve of random forest model construction with a residual value of 1. (**B**) The reverse cumulative distribution curve plot to show the residual distribution of the random forest model.

*Ralstonia* and *Rhizobiaceae* were observed in both predictions. However, the decrease in the count of these microbiomes contributed to the control sample prediction. The prediction probability confidence of the model on the cervical cancer sample is 0.07, and the control sample is 0.85, with class label values 0 and 1, respectively. Figure 12 depicts the ROC curve of the random forest model at the top, with a score of 1. The reverse cumulative distribution curve at the bottom indicates that most residuals fall below 0.1. This phenomenon occurs when the dataset contains many features, assigning varying contributions to every feature.

## 4. Discussion

Characterization of the microbiome is essential to untangle the disease etiology. Microbial dysbiosis is a crucial factor associated with disease dynamics, also evident in accurate diagnosis of the condition. This study analyzed the vaginal microbiome of 65 cervical cancer and 54 healthy samples to discern microbial pathogenicity. The taxon abundance assessment at different levels determined unique microbial patterns exhibiting clear discrimination between the case and control groups. *Firmicutes*, *Actinobacteria*, and *Proteobacteria,* are abundant at the Phylum level. *Lactobacillus* genera are elevated when compared to *Gardnerella* and *Streptococcus*. In much literature, the influence of *Lactobacillus* on cervical cancer is reported [42,43]. *Lactobacillus iners* showed higher abundance in cervical samples over control (Figure 4). The oncogenic nature of *Lactobacillus iners* in cervical cancer was delineated in a microbial study [44]. Other abundant species, such as *Prevotella timonensis* [45,46], *Gardnerella vaginalis* [47], and *Streptococcus anginosus* [48], confirmed microbial pathogenicity.

The diversity and richness analysis identified a decline in the cervical cancer microbial community, calculated by Shannon and Chao index. The Bray–Curtis distance measure was used to quantify the compositional dissimilarity of the microbiome, visualized using PCoA. The plot displayed a distinct cluster pattern among the vaginal microbial communities of cervical cancer and healthy control groups with *p*-value: 0.001, R2: 0.027, and F-value: 3.269 (Figure 6). LEfSe predicted enriched taxonomical units at a different level. *Lactobacillus iners* ranked top, followed by *Pseudomonas*, *Streptococcus*, and *Enterococcus*, describing the pathogenic association with cervical cancer. *Proteobacteria*, *Rhizobiaceae*, and *Bacteriodota* were highly enriched in the control group.

The differentially expressed taxa were calculated to perform disease-functional enrichment of microbiomes. The disease association of the enlisted taxa reported aerobic vaginitis, bacterial vaginosis, and chlamydia. Prolonged exposure to the pathogenic bacterial environment increases the risk of developing cervical cancer [49]. Another dimension of this study scrutinized the influence of each microbe contributing to the discrimination of cervical cancer and control samples. It examined the importance of each feature and its impact on prediction through SHAP values. The dataset was trained with a random forest ensemble classification algorithm. The prediction result of the model was interpreted using the SHAP algorithm. Increased *Ralstonia* impacted the prediction of the sample as cervical cancer with a higher probability (0.056) [50].

Conversely, the highly pathogenic taxa, *Streptococcus* [51], has a minor abundance contributing to the prediction (0.058) of the control sample, followed by *Ralstonia* (0.057). The reverse cumulative distribution curve indicates that the features lie below 0.1, impacting the predictions (Figure 12). The lesser value is due to many features (594) in the database. This study unveiled many potential pathogenic vaginal microbiomes causing a detrimental effect on individuals. Meanwhile, there exist many factors involved in the disease condition. Multi-omic studies on cervical cancer will further broaden the understanding of the disease etiology. Clinical informatics, combined with artificial intelligence, makes personalized medicine possible in the near future to treat complex diseases through effective mechanisms.

## 5. Conclusions

This study identified the dominance of *Lactobacillus iners* species in the vaginal microbiome of cervical cancer samples. The imbalance in microbial distribution is observed during α diversity analysis. *Lactobacillus*, *Gardnerella*, *Pseudomonas,* and *Enterococcus* are abundant at the genus level in cervical cancer. The microbiome disease association enrichment detects increased susceptibility with aerobic vaginitis, bacterial vaginosis, and chlamydia. These diseases have a direct coalition with cervical cancer and other severe vaginal infections. The discriminative evidence to classify healthy and cervical cancer group samples is deliberated with the SHAP model. The explainable approach identifies *Ralstonia* as a microbial predictor marker. The increased composition of *Ralstonia* impels the model to predict the sample as cervical cancer. Though *Ralstonia* is not reported as highly prevalent in cervical cancer, this inference unveils the decisive characteristics of the marker. Thus, the current findings invigorate the development of probiotics as targeted therapeutics for effective treatment. The following limitation is identified and reported in the present work. This study delineates the microbiome information of a single dataset, and though it is valid, a comparative analysis cannot be conducted. In the future, this study could be further extended by adding more datasets to demonstrate and benchmark the results, thereby ensuring in-depth validation of the findings.

## Data Availability

The data are available with the corresponding author AEA and GPDC. The code is available in the following github link: https://github.com/karthiksekaran/microbiome-AI, accessed on 4 December 2022.

## Supplementary Materials

The following supporting information can be downloaded at: https://www.mdpi.com/article/10.3390/genes14040936/s1, Table S1: Functional Enrichment Analysis, Supplementary file: Repeated k-fold cross-validation results.
