# Supplementary material for: Unraveling the Dysbiosis of Vaginal Microbiome to Understand Cervical Cancer Disease Etiology—An Explainable AI Approach
Source: Genes (Basel). 2023 Apr 18;14(4):936. doi: 10.3390/genes14040936 (PMC10137380; doi:10.3390/genes14040936)
Supplement: Supplementary file 1 [file genes-14-00936-s001.zip › genes-2272066-supplementaryfile .pdf]

Result Summary: Code with Outputs

```
# evaluate a random forest model using repeated k-fold cross-validation
from numpy import mean
from numpy import std
from sklearn.datasets import make_classification
from sklearn.model_selection import RepeatedKFold, cross_validate
from sklearn.model_selection import cross_val_score
from sklearn.linear_model import LogisticRegression
from sklearn.ensemble import RandomForestClassifier
# create dataset
import pandas
data = pandas.read_csv("cervical-modeling.csv")

X = data.iloc[:,:-1]
y = data.iloc[:,-1]

from sklearn import preprocessing
cv = RepeatedKFold(n_splits=10, n_repeats=5, random_state=1)
# create model
fr = RandomForestClassifier()
# evaluate model
#scores = cross_val_score(fr, X, y, scoring='accuracy', cv=cv, n_jobs=-1)
scores = cross_validate(fr, X, y, scoring='accuracy', cv=cv,
return_estimator=True )
# report performance
scores
```

Output:

```
{'fit_time': array([0.17345953, 0.30371141, 0.1110363 , 0.09734368,
0.17350817,
        0.18749547, 0.15978193, 0.14586473, 0.15343857, 0.13212061,
        0.12495637, 0.12490106, 0.17328167, 0.1736238 , 0.11151195,
        0.12519979, 0.09729004, 0.10423589, 0.10427928, 0.10406303,
        0.10431886, 0.0976243 , 0.09720755, 0.18046546, 0.15983033,
        0.17378521, 0.15276527, 0.13907123, 0.13893175, 0.13902807,
        0.13864064, 0.15291071, 0.16179061, 0.14871764, 0.15010834,
        0.15442991, 0.15718937, 0.13781261, 0.14408517, 0.14207697,
        0.15301275, 0.15224051, 0.14404821, 0.21784496, 0.2705338 ,
        0.27771688, 0.25702167, 0.25691867, 0.24995995, 0.2570014 ]),
 'score_time': array([0.01563358, 0.01394057, 0.01391459, 0.00683832,
0.02091599,
        0.0139792 , 0.02072573, 0.01397491, 0.02056003, 0.01405406,
        0.02076554, 0.01404166, 0.02789807, 0.01396799, 0.0135138 ,
        0.01396871, 0.0136528 , 0.00686097, 0.00704408, 0.01374578,
        0.00689292, 0.00692248, 0.0140624 , 0.02765703, 0.01365042,
        0.0207386 , 0.01382923, 0.0139389 , 0.013762  , 0.01392341,
        0.01411128, 0.01375031, 0.01352429, 0.01962209, 0.01442814,
        0.01444411, 0.01437926, 0.01352572, 0.01433611, 0.01634073,
        0.01633072, 0.0147953 , 0.01535606, 0.02811646, 0.02782512,
        0.02777004, 0.0276885 , 0.02783275, 0.02773595, 0.02085686]),
 'estimator': [RandomForestClassifier(),
  RandomForestClassifier(),
  RandomForestClassifier(),
  RandomForestClassifier(),
  RandomForestClassifier(),
  RandomForestClassifier(),
  RandomForestClassifier(),
  RandomForestClassifier(),RandomForestClassifier(),
  RandomForestClassifier(),
```

```
  RandomForestClassifier(),
  RandomForestClassifier(),
  RandomForestClassifier(),
  RandomForestClassifier(),
  RandomForestClassifier(),
  RandomForestClassifier(),
  RandomForestClassifier(),
  RandomForestClassifier(),
  RandomForestClassifier(),
  RandomForestClassifier(),
  RandomForestClassifier(),
  RandomForestClassifier(),
  RandomForestClassifier(),
  RandomForestClassifier(),
  RandomForestClassifier(),
  RandomForestClassifier(),
  RandomForestClassifier(),
  RandomForestClassifier(),
  RandomForestClassifier(),
  RandomForestClassifier(),
  RandomForestClassifier(),
  RandomForestClassifier(),
  RandomForestClassifier(),
  RandomForestClassifier(),
  RandomForestClassifier(),
  RandomForestClassifier(),
  RandomForestClassifier(),
  RandomForestClassifier(),
  RandomForestClassifier(),
  RandomForestClassifier(),
  RandomForestClassifier(),
  RandomForestClassifier(),
  RandomForestClassifier(),
  RandomForestClassifier(),
  RandomForestClassifier(),
  RandomForestClassifier(),
  RandomForestClassifier(),
  RandomForestClassifier(),
  RandomForestClassifier(),
  RandomForestClassifier()],

'test_score': array([1.        , 1.        , 0.83333333, 1.        , 1.
,
        1.        , 1.        , 1.        , 0.91666667, 0.90909091,
        0.91666667, 0.91666667, 1.        , 1.        , 1.        ,
        0.91666667, 1.        , 1.        , 1.        , 1.        ,
        0.91666667, 1.        , 1.        , 0.91666667, 0.91666667,
        1.        , 0.91666667, 1.        , 1.        , 0.90909091,
        1.        , 1.        , 0.91666667, 1.        , 1.        ,
        1.        , 0.91666667, 1.        , 1.        , 1.        ,
        1.        , 0.9166
```

```
mean(scores['test_score'])
```

0.9713636363636362

```python
# evaluate a random forest model using k-fold cross-validation
from numpy import mean
from numpy import std
from sklearn.datasets import make_classification
from sklearn.model_selection import RepeatedKFold
from sklearn.model_selection import cross_val_score
from sklearn.linear_model import LogisticRegression
from sklearn.ensemble import RandomForestClassifier
# create dataset
import pandas
data = pandas.read_csv("cervical-modeling.csv")
from sklearn.model_selection import KFold
X = data.iloc[:,:-1]
y = data.iloc[:,-1]

from sklearn import preprocessing
cv = KFold(n_splits=10, random_state=None)
# create model
model = RandomForestClassifier()
# evaluate model
scores = cross_val_score(model, X, y, scoring='accuracy', cv=cv, n_jobs=-1)
# report performance
print('Accuracy: %.3f (%.3f)' % (mean(scores), std(scores)))
```

Accuracy: 0.962 (0.092)
